# IL-4, IL-7, IL-9, NT, NRP1 May Be Useful Markers in the Diagnosis of Endometrial Cancer

**DOI:** 10.3390/biom14091095

**Published:** 2024-09-01

**Authors:** Mateusz Kozłowski, Dominika Borzyszkowska, Natalia Lerch, Agnieszka Turoń-Skrzypińska, Marta Tkacz, Jerzy Lubikowski, Maciej Tarnowski, Iwona Rotter, Aneta Cymbaluk-Płoska

**Affiliations:** 1 Department of Reconstructive Surgery and Gynecological Oncology, Pomeranian Medical University in Szczecin, al. Powstańców Wielkopolskich 72, 70-111 Szczecin, Poland; 2 Department of Medical Rehabilitation and Clinical Physiotherapy, Pomeranian Medical University in Szczecin, Żołnierska 48, 71-210 Szczecin, Poland; 3 Department of Physiology in Health Sciences, Faculty of Health Sciences, Pomeranian Medical University in Szczecin, Żołnierska 54, 70-210 Szczecin, Poland

**Keywords:** endometrial cancer, diagnosis, biomarker, IL-4, IL-7, IL-9, IL-10, neurotensin, thrombospondin-2, neuropilin 1

## Abstract

The search for novel endometrial cancer diagnostic biomarkers is pertinent. The purpose of this study was to determine if IL-4, IL-7, IL-9, IL-10, NT, TSP-2, and NRP1 could be used as novel, helpful markers for the detection of endometrial cancer. Ninety-three women diagnosed with endometrial cancer (EC) and sixty-six patients with noncancerous endometrial lesions (NCEL) were included in this study. ELISA was used to measure the concentrations of the proteins tested. Median serum levels of IL-4, IL-7, IL-9, NT, and NRP1 were significantly higher in the EC group compared with NCEL. The cut-off level of IL-4 was set at 802.26 pg/mL with a sensitivity of 83.87% and a specificity of 50% (AUC = 0.7, *p* = 0.000023). The cut-off level of IL-7 was set at 133.63 ng/L with a sensitivity of 96.77% and a specificity of 75.76% (AUC = 0.91, *p* < 0.000001). The cut-off level of IL-9 was set at 228.79 pg/mL with a sensitivity of 69.89% and a specificity of 81.82% (AUC = 0.8, *p* < 0.000001). The cut-off level of NT was set at 275.43 pmol/L with a sensitivity of 94.62% and a specificity of 59.09% (AUC = 0.83, *p* < 0.000001). The cut-off level of NRP1 was set at 30.37 ng/mL with a sensitivity of 81.72% and a specificity of 57.58% (AUC = 0.71, *p* = 0.000004). This study suggests the clinical utility of IL-4, IL-7, IL-9, NT, and NRP1 in the diagnosis of endometrial cancer. Nevertheless, these biomarkers may also have prognostic or predictive value, which should be tested in future studies.

## 1. Introduction

Endometrial cancer is the most common gynecological malignancy and the fourth most prevalent malignancy in women [1,2]. The incidence of endometrial cancer is steadily increasing worldwide [3]. In 2020, it was observed that 417,367 women globally developed endometrial cancer, with Poland having 9869 cases, representing the highest global incidence rate [4]. The risk of endometrial cancer increases with age. Most cases occur in postmenopausal women, typically between the sixth and seventh decades of life [5,6]. Approximately 90% of women with endometrial cancer experience vaginal bleeding. The presence of postmenopausal bleeding prompts patients to undergo diagnostic procedures, including transvaginal ultrasound, endometrial biopsy, hysteroscopy, and often curettage of the uterine cavity [7]. To date, there is currently no evidence supporting population-based screening for endometrial cancer in asymptomatic women at average risk [8,9]. Even though symptoms of endometrial cancer appear at an early stage of the disease, identifying risk factors and biomarkers useful in diagnosing this malignancy is crucial for early diagnosis. So far, numerous risk factors for endometrial cancer have been documented, with obesity being a major contributor. Women with a BMI > 30 have a significantly higher mortality rate compared with those with a normal body weight [7,10]. Other risk factors include exposure to estrogens, diabetes, hypertension, tamoxifen therapy, early menarche, late menopause, and low parity [5]. Many of these risk factors have strong proinflammatory effects. Increasingly, a strong correlation is being observed between inflammation and cancer [11].

While existing diagnostic procedures have excellent accuracy, there are difficulties due to their intrusive nature and accompanying pain [7,12]. The search for a new, noninvasive, and affordable diagnostic instrument is essential to reduce needless curettage and create new therapeutic approaches as well as preventive strategies. Peripheral blood samples have shown promise as prospective biomarker repositories because they are easily accessible and generally noninvasive. The sensitivity and specificity of currently available serum tumor markers, such as HE4 and CA125, are still unclear, despite their involvement in the diagnosis of EC [13,14,15]. The examination of new biomarkers can contribute to a better understanding of the underlying mechanism of endometrial cancer’s development and progression. The proteins examined in this study are involved in key processes related to tumor formation, such as inflammatory responses [16], angiogenesis [17,18], cell proliferation [19,20], and apoptosis inhibition [21,22]. Given the scarcity of data on these proteins in endometrial cancer, this study aims to evaluate the diagnostic potential of preoperative serum concentrations of IL-4, IL-7, IL-9, IL-10, NT, TSP-2, and NRP1.

Interleukin-4 (IL-4) is an immunomodulatory cytokine that promotes the differentiation of T lymphocytes towards the Th2 phenotype, which in turn supports humoral response and IgE antibody production [23,24]. Due to its antiangiogenic and immunosuppressive properties, it can influence cancer processes [13]. In endometrial cancer, IL-4’s role as a potential biomarker remains to be fully understood. Some studies associate higher levels of IL-4 with more advanced cancers, suggesting its potential as a prognostic marker [13,25,26]. On the other hand, IL-4 has been linked to reduced cell proliferation in colon, kidney, and breast cancers, highlighting its complex role in tumor biology [13,27]. Interleukin-7 (IL-7) is crucial for T and B cell development [28]. It demonstrates antitumor activity by enhancing tumor eradication [21]. However, IL-7’s protumor effects, including the inhibition of apoptosis and promotion of neoplastic growth via *BCL* gene regulation, have also been noted [21,22]. The limited research on IL-7 in endometrial cancer suggests a need to explore its role as a potential diagnostic marker. Interleukin-9 (IL-9) is another physiologically secreted cytokine that impacts hematopoiesis, mast cells, smooth muscle cells, and respiratory epithelial cells. It plays a role in various cancers, including lung, breast, and thyroid cancer, by promoting cell growth [19]. Studies on the impact of IL-9 on endometrial cancer have highlighted the significant infiltration of IL9+ cells and overexpression of IL9R tumor tissues [29]. These findings suggest that IL-9 may have a crucial diagnostic role in identifying endometrial cancer and a prognostic role in evaluating disease severity and progression. Interleukin-10 (IL-10) is known for its ability to regulate immune responses, including the inhibition of T lymphocyte activity [20]. This suppression can potentially lead to the proliferation of cancer cells [30]. In endometrial cancer, IL-10 has been shown to promote epithelial–mesenchymal transition (EMT) and vascular mimicry, which are processes associated with tumor progression, suggesting a possible role in assessing tumor aggressiveness and patient outcomes [31]. 

Neurotensin (NT) is a regulatory protein involved in gastrointestinal motility, lipid metabolism, and neuromodulation [32,33,34]. NT increases the expression of HER2, HER3, and EGFR, indicating its protumor activity [32,34,35]. Research on neurotensin has shown its protumorigenic effects on hormone-dependent tumors, and it has been found that the NT complex and its receptor 1 contribute to cancer progression [35,36]. This suggests the utility of NT as a predictor of metastatic potential in endometrial cancer. Thrombospondin-2 (TSP-2) is a glycoprotein involved in angiogenesis, extracellular matrix formation, and the regulation of proliferation [18,37]. Higher expression of TSP-2 has been observed in endometrial cancer, particularly in the cervical space and lymphatic vessels, suggesting its potential as a prognostic marker [18]. Neuropilin 1 (NRP1) is a transmembrane protein that mediates cytokine signaling and is associated with increased VEGF expression, promoting angiogenesis and tumor growth [38,39]. Additionally, NRP1’s role as a co-receptor for TGF-β1 may facilitate processes linked to tumor progression, such as cell proliferation and metastasis [38,40]. Given the above, NRP1 could not only serve as a diagnostic marker but also as a predictor of metastatic potential in endometrial cancer.

The aim of this study was to determine the clinical significance of the proteins studied. First, we compared the serum concentrations of the proteins in patients with noncancerous endometrial lesions (NCEL) and endometrial cancer (EC). Finally, we evaluated the significance of IL-4, IL-7, IL-9, IL-10, NT, TSP-2, and NRP1 in the diagnosis of endometrial cancer. Due to the lack of appropriate and current research, this study will expand the knowledge regarding these proteins in endometrial cancer.

## 2. Materials and Methods

### 2.1. Study Design

This study was a retrospective noninterventional one. Patients having abrasions for abnormal uterine bleeding were included in the study. Oncologic surgery was then appropriate for patients who had been diagnosed with endometrial cancer based on histopathology. There was a control group without endometrial cancer and a research group with endometrial cancer. Subgroups within the study group were created according to the histological subtype, grading, and clinical stage (staging). Both the study and control groups had their serum levels of the proteins under investigation measured.

This study was conducted in accordance with the Declaration of Helsinki and approved by the Bioethics Committee of the Pomeranian Medical University in Szczecin (protocol code KB-0012/77/12 on 13 October 2012).

### 2.2. Participation in the Study

Those receiving treatment for perimenopausal uterine hemorrhage were included in this study. Lack of patient consent, inadequate patient data, treatment history for another malignancy, pelvic inflammatory illness, histological diagnosis of uterine malignancy other than cancer, imbalanced chronic disorders, and autoimmune diseases were the exclusion criteria for this study. In the end, 159 women met the study’s eligibility requirements. Based on their weight and height, the patients’ body mass index (BMI) was determined at the start of the trial. The formula used to determine the BMI was BMI = weight [kg]/height^2^ [m^2^]. The patients were classified into two subgroups based on the results: those with normal weight (BMI 18.5–24.9) and those who were overweight or obese (BMI > 30). Every patient also had their blood pressure measured. We separated the patients into groups with (>140/90) and without hypertension based on the results. Furthermore, we evaluated the occurrence of type 2 diabetes (DM2) based on the medical history of the patients. The power of the Mann–Whitney U test was at a very good level in most of the cases analyzed. The exception is the evaluation of the concordance of distributions for BMI in the groups of patients studied and in terms of BMI in the group of patients with a BMI below 25. The group characteristics are shown in Table 1 and Table 2.

### 2.3. Laboratory Analysis

The preoperative serum samples were aliquoted and frozen to a temperature of –80 °C. IL-4, IL-7, IL-9, IL-10, NT, TSP-2, NRP1 levels were determined by an enzyme-linked immunosorbent assay (Shanghai Sunredbio Technology Co., Ltd., Shanghai, China, Cat No. 201-12-0093C, 201-12-1949C, 201-12-0089C, SRB-T-83474, 201-12-1319, 201-12-2386, 201-12-4380, respectively) according to the manufacturer’s protocols [41,42]. The specific steps were as follows: the standard of IL-4, IL-7, IL-9, IL-10, NT, TSP-2, NRP1 was diluted to the corresponding concentration series, then the samples, antibodies, and 50 µL of Streptavidin-HRP were added to each well and incubated for 60 min at 37 °C. After washing, the chromogenic agent was used, and the samples were placed in the dark and incubated for 10 min at 37 °C with gently mixed. In the next step, the stop solution was added, and the optical density (OD) under 450 nm wavelength was measured using a microplate reader (Infinite F50, Tecan, Männedorf, Switzerland) for up to 15 min. According to standard concentrations and corresponding OD values, the standard curve linear regression equation was calculated and corresponding sample concentrations were reported in pg/mL, ng/L, pmol/L, µg/mL, and ng/mL.

### 2.4. Statistical Calculations

Statistica 13.3.0 was used to conduct the statistical calculation. The gathered research material was assessed using the nonparametric Mann–Whitney U test of significance, correlation analysis, and statistical description. Additionally, a ROC-curve-based diagnostic test was employed. The asymmetry coefficient, minimum and maximum values, arithmetic mean, and median were used to perform the statistical description in the first stage. The normality of the distribution plot and the Shapiro–Wilk test were used to evaluate the distribution of variables. The values of descriptive parameters characterizing the distributions of the variables were also determined. A nonparametric significance test called the Mann–Whitney U test was employed to compare the distributions because the other variables were stated on a nominal scale and the distributions of the quantitative variables were not normal. When examining correlations between quantitative variables, Spearman’s rank-order correlation coefficient (test of significance for Spearman’s rank-order correlation coefficient) was utilized as a nonparametric measure. This paper also evaluated the power of the significance tests used. The power of the tests was checked to detect the existence of an effect in the form of correlation or difference between mean values. The power analysis was carried out a posteriori, that is, after the data were collected. The following information was used to detect the power of the test: sample size, significance level (α = 0.05), and effect size. A test power of at least 0.8 (80%) is sought, meaning that the test has an 80% chance of detecting an effect if it actually exists. Conversely, variables representing protein concentrations and variables expressed on a nominal scale were correlated using Pearson’s C contingency coefficient, which is based on the χ^2^ statistic (chi square). To differentiate individuals with the feature from those without, a diagnostic test based on the ROC curve—more specifically, the area under the ROC curve—was employed. This was performed using DeLong’s nonparametric technique.

## 3. Results

### 3.1. Serum Concentrations of the Studied Proteins in the Study Group and the Control Group

First, we determined the serum levels of the proteins studied in the study group and the control group. We then compared the median serum concentrations of the markers studied. We found significantly higher concentrations of IL-4, IL-7, IL-9, NT, NRP1 in the EC group compared with NCEL. We also found significantly lower concentrations of IL-10 in the EC group compared with the NCEL group. The comparison of concentrations for TSP-2 was not significant (*p* = 0.6787), so this protein was not included in further statistical calculations. The power of the Mann–Whitney U test was at a very good level in most of the cases analyzed. However, low power is shown by the test performed for TSP-2 for the groups of female patients studied. Detailed data are shown in Table 3.

### 3.2. Correlations between Studied Proteins

In the next step, the correlations of the proteins studied were investigated. A significant positive correlation was found between IL-4 and IL-7 (r = 0.395, *p* = 0.0000001), IL-4 and IL-9 (r = 0.396, *p* = 0.0000001), IL-4 and NT (r = 0.394, *p* = 0.0000001), IL-4 and NRP1 (r = 0.304, *p* = 0.0001), IL-7 and IL-9 (r = 0.628, *p* = 0.0000001), IL-7 and NT (r = 0.529, *p* = 0.0000001), IL-7 and NRP1 (r = 0.259, *p* = 0.000982), IL-9 and NT (r = 0.629, *p* = 0.0000001), IL-9 and NRP1 (r = 0.264, *p* = 0.000788), NT and NRP1 (r = 0.251, *p* = 0.001391). A significant negative correlation was found between IL-7 and IL-10 (r = −0.420, *p* = 0.0000001), IL-9 and IL-10 (r = −0.229, *p* = 0.003764), IL-10 and NT (r = −0.348, *p* = 0.000007), IL-10 and NRP1 (r = −0.232, *p* = 0.003313). The correlation between IL-10 and IL-4 was nonsignificant (r = −0.149, *p* = 0.061206). Analyzing the power of the test for Spearman’s rank correlation coefficient, a very weak power is noted for the correlation between IL-4 and IL-10. A test power of less than 0.8 was observed when verifying the relationship between IL-4 and IL-10, IL-7 and NRP1, IL-9 AND IL-10, IL-9 and NRP1, IL-10 and NRP1, NT and NRP1. All data are shown in Table 4.

### 3.3. Receiver Operating Characteristic (ROC) Curve for Using IL-4, IL-7, IL-9, IL-10, NT, NRP1 Distinguishing between Endometrial Cancer and Noncancerous Endometrial Lesions

**Figure 1:**
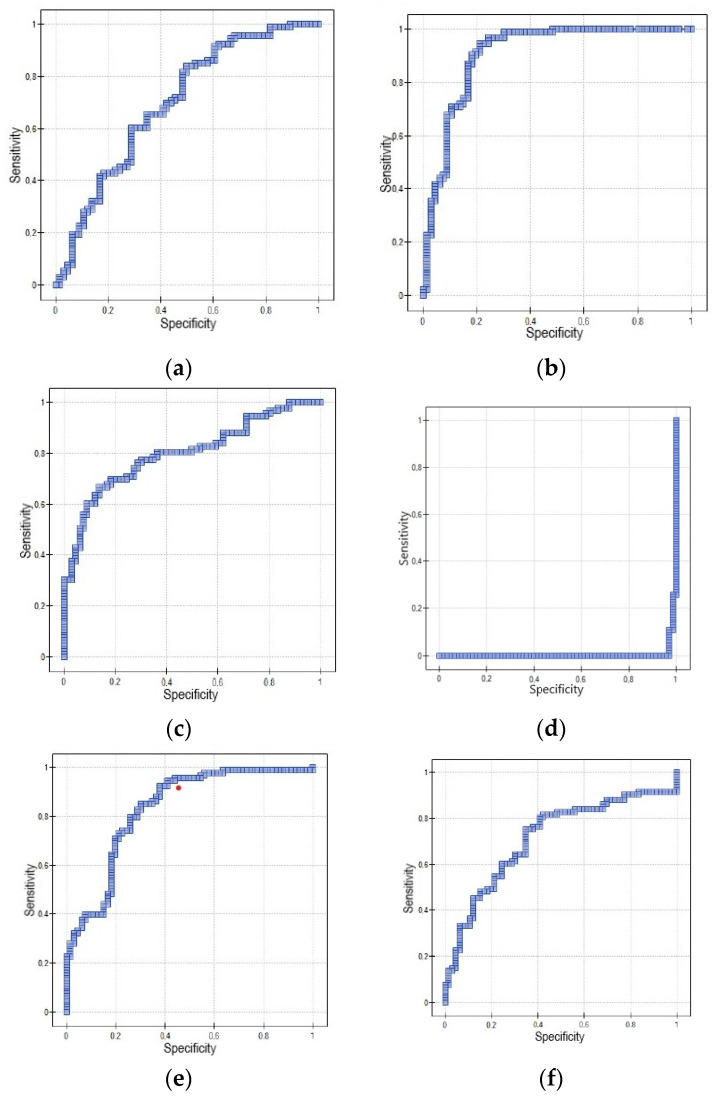
ROC Curve using IL-4 (**a**), IL-7 (**b**), IL-9 (**c**), IL-10 (**d**), NT (**e**), and NRP1 (**f**) distinguishing between EC and NCEL.

The diagnostic utility of the tested proteins between EC and NCEL was investigated using the ROC curve. The cut-off level of IL-4 was set at 802.26 pg/mL with a sensitivity of 83.87% and a specificity of 50% (AUC = 0.7, *p* = 0.000023). The cut-off level of IL-7 was set at 133.63 ng/L with a sensitivity of 96.77% and a specificity of 75.76% (AUC = 0.91, *p* < 0.000001). The cut-off level of IL-9 was set at 228.79 pg/mL with a sensitivity of 69.89% and a specificity of 81.82% (AUC = 0.8, *p* < 0.000001). The cut-off level of IL-10 was set at 187.4 pg/mL with a sensitivity of 100% and a specificity of 0% (AUC = 0.006, *p* < 0.000001). The cut-off level of NT was set at 275.43 pmol/L with a sensitivity of 94.62% and a specificity of 59.09% (AUC = 0.83, *p* < 0.000001). The cut-off level of NRP1 was set at 30.37 ng/mL with a sensitivity of 81.72% and a specificity of 57.58% (AUC = 0.71, *p* = 0.000004). The detailed data are shown in Table 5 and Figure 1.

## 4. Discussion

Despite being the most common malignant gynecological tumor in women, endometrial cancer (EC) is not as well-studied as cervical and ovarian cancers, and its incidence continues to rise. To date, no reliable biomarker for the diagnosis of endometrial cancer has been identified, highlighting the importance of continued research in this field. Our study aimed to determine whether IL-4, IL-7, IL-9, IL-10, NT, TSP-2, and NRP1 could be diagnostic markers for endometrial cancer in women. The results obtained show that the median serum concentrations of IL-4, IL-7, IL-9, NT, and NRP1 were higher in women with EC compared with those with NCEL. The evaluation of IL-10’s utility revealed an AUC of less than 0.5 for this cytokine, and its specificity was 0%. Moreover, the median serum concentration of IL-10 was higher in the NCEL group compared with those with EC. Therefore, interleukin-10 was excluded as a potential diagnostic marker for EC. In the case of TSP-2, the difference in serum concentration was not significant, with a *p*-value greater than 0.05, and therefore, it was also disqualified as a diagnostic marker. Although the TSP-2 has been observed to play an important role in other cancers, regulating angiogenesis and extracellular matrix, it lacks significance in our study. This suggests that its role in EC may be less prominent or context-dependent. Variations in study groups, patient demographics, or assay sensitivity could contribute to these findings. Further research is needed to elucidate TSP-2’s role in endometrial cancer and determine if there are specific subgroups where it might be relevant.

Interleukin-4 is an immunomodulatory cytokine that plays a dual role in the pathogenesis of tumor development, exhibiting both antiangiogenic and immunosuppressive properties, which may facilitate tumor progression by creating an appropriate immune environment [13,43,44]. IL-4’s functional and biological roles have been extensively studied across various cancer types. In breast, kidney, and colorectal cancers, it has been shown to have an inhibitory effect on cell proliferation, while in lung cancer, it has been found to enhance the proliferation of myeloid cells [13,45]. Bermúdez-Morales et al. found high local expression of IL-4 in biopsies from patients with precancerous changes and cervical cancer, simultaneously suggesting the induction of a local immunosuppressive state [46]. Various studies have shown that IL-4 can function as a diagnostic as well as a prognostic marker. Ramspott et al. demonstrated that the presence of IL4I1+ cells was associated with sentinel lymph node invasion and more advanced stages of melanoma [47]. The study conducted by Jincheng Ma et al. aimed to evaluate the role of IL-4 detection in endometrial cancer screening and tumor progression. In their results, they identified low interleukin-4 levels as an independent risk factor for EC in a group of patients with AUB [13]. Although in recent years there has been growing interest in the potential use of Interleukin-4 as a marker in the serum of patients with various types of cancers, its role in endometrial cancer remains controversial. Our study showed that the serum IL-4 concentration in patients with EC was higher compared with patients with NCEL. This result is consistent with the statement that IL-4 may contribute to cancer progression by supporting an immunosuppressive response, promoting the development and invasion of cancer cells. Wieder-Huszla et al. assessing cytokine levels in patients with ovarian cancer and endometrial cancer, found that IL-4 levels were higher in the group of patients with ovarian cancer than in those with endometrial cancer. However, in the group of patients with EC, the IL-4 level was noticeably higher compared with other analyzed cytokines, although the difference was statistically insignificant [24]. All these studies indicate an undeniable role in the pathogenesis of tumor development, but despite promising results, further research is necessary to confirm the role of IL-4 as a marker in endometrial cancer. 

Interleukin-7 is a cytokine known for its crucial role in the development and homeostasis of T and B cells. It is also responsible for neoplastic growth and inhibition of apoptosis [21,22]. Some studies attribute IL-7 with the potential to act as an angiogenic enhancer [48]. Although available studies on IL-7 in endometrial cancer are limited, the literature contains data on the assessment of this cytokine in other cancers. In cervical cancer, IL-7 levels were significantly elevated, especially in the invasive stages, indicating its potential involvement in tumor progression [48]. Similarly, in ovarian cancer, increased IL-7 levels have been linked to disease progression and poor clinical outcomes [49,50]. In breast cancer, elevated IL-7 levels have been associated with enhanced tumor growth and poor prognosis [51]. These studies suggest that IL-7 may function not only as a diagnostic marker but also as a prognostic marker. In our study, we observed elevated levels of IL-7 in the serum of patients with endometrial cancer compared with the control group. Chopra et al. also noted an increase in IL-7 levels in the EC group. Moreover, these studies suggest a probable link between elevated IL-7 levels and more advanced stages of the disease, as well as poorer prognosis [52]. Our findings contribute to the growing body of evidence suggesting that IL-7 could be a useful biomarker for the diagnosis of endometrial cancer. Additionally, the association between higher IL-7 levels and advanced disease stages in EC suggests a prognostic role for this cytokine, similar to its role in other cancers.

IL-9 is a cytokine involved in the regulation of inflammatory response and the modulation of immune cell function. It plays a complex role in cancer, exhibiting both protumorigenic and antitumorigenic effects depending on the cancer type [19]. For instance, in lung cancer, IL-9 does not directly increase cancer cell growth but modulates cell proliferation and inhibits apoptosis, thereby supporting tumor survival [19]. In breast and thyroid cancer, IL-9 has been implicated in promoting cell growth and contributing to tumor progression [19]. In endometrial cancer, studies by Tong et al. have highlighted significant infiltration of IL-9+ cells and overexpression of its receptor in tumor tissues, suggesting that IL-9 may facilitate tumor development through its impact on the microenvironment and immune modulation [29]. Zang et al. presented a correlation between IL9+ lymphocyte and patient survival, along with the expression of progesterone receptors in EC [53]. Il-9 role as a diagnostic and prognostic marker varies across different cancers. Research by Habel et al. revealed that IL-9 can be used for early detection of epithelial ovarian cancer [54]. Fu X et al. evaluated the expression of IL-9 and IL-22 in the peripheral blood of patients with endometrial cancer, showing significant differences in cytokine expression between the endometrial cancer group and the control group. They found that the levels of IL-9 and IL-22 were higher in both the EC group and the atypical hyperplasia (AH) group compared with the control group, suggesting their potential role in the pathogenesis and progression of precancerous and cancerous changes. They also observed statistically significant differences in the expression of IL-9 and IL-22 between the EC, AH, and control groups, with the highest levels of these cytokines noted in the EC group and the lowest in the control group, highlighting their value as diagnostic and prognostic biomarkers in endometrial cancer [55]. The increase in IL-9 expression may be associated with the promotion of inflammatory and immunosuppressive processes, which favor tumor development and progression. Our findings are consistent with previous findings, demonstrating a notable increase in IL-9 expression in serum samples from EC patients. This alignment across different sample types reinforces the potential of IL-9 as a diagnostic marker for endometrial cancer. Nonetheless, further research is necessary to fully elucidate the mechanisms by which IL-9 contributes to endometrial cancer pathogenesis and to validate its use as a clinical biomarker.

Neurotensin is known for its regulatory role in the nervous and gastrointestinal system, but its function extends to the modulation of cancer cell behavior. NT plays a significant role in neoplastic progression, including the proliferation of the prostate, colon, pancreas, and lung cancer cells [56]. It also prevents apoptosis, including breast cancer. As a result, elevated NT levels, occurring in tissues, can play a major role in tumor proliferation [57]. The available studies suggest that NT and its receptor NTSR1 may serve not only as diagnostic markers but importantly as prognostic markers. For instance, in breast cancer, elevated levels of NT have been associated with increased tumor aggressiveness and poor survival rates [56,58]. Similarly, in pancreas cancer, increased expression levels of NT and its receptor were found in highly malignant sublines. Further analysis revealed that higher levels were present in more advanced cancer and that high NSTR1 concentrations were correlated with poor prognosis [59]. The research on neurotensin in endometrial cancer is limited. Our study results indicate that in the serum of patients with EC, the concentration of neurotensin was higher compared with the NCEL control group. These findings are consistent with previous reports in the literature suggesting that NT plays a crucial role in the pathogenesis and progression of cancer. Immunohistochemical studies conducted by Agopiantz et al. demonstrated that the expression of NT and NTSR1 is significantly increased in endometrial cancer compared with the control group. Furthermore, a negative correlation has been shown between NTSR1 activity and overall survival, as well as progression-free survival in EC patients, thereby highlighting the role of NT in tumor aggressiveness [35]. NT’s elevated serum levels in EC patients, along with its established roles in other cancers, highlight its importance as both a diagnostic and prognostic biomarker. Further research is needed to fully understand the mechanisms of NT in endometrial cancer.

Neuropilin 1 is a transmembrane protein that plays a significant role in the pathogenesis of tumor development. It acts as a co-receptor for various signaling molecules, including vascular endothelial growth factor (VEGF) and transforming growth factor- beta 1(TGF-β1) [38,39]. By binding to VEGF, NRP1 promotes angiogenesis, which is crucial for tumor growth and metastasis across multiple cancer types [60]. Additionally, NPR1’s role as a co-receptor for TGF-β1 facilitates cell proliferation and metastasis, further implicating NRP1 in cancer development and spread [61]. For instance, in non-small-cell lung cancer (NSCLC), NRP1 was significantly increased and functioned as a vital promoter of metastasis [62]. In terms of diagnostic and prognostic applications, NRP1 has shown promise across various cancers. As an example, Cao et al. analyzed the expression of NRP1 in gastric cell tissue and it was higher in more advanced stages compared with the group of early stage [63]. Similarly, Oplawski et al. demonstrated that NRP1 expression was elevated in tissue samples from endometrial cancer patients compared with the control group. Furthermore, NRP1 expression was strongly correlated with histological malignancy. In G2 and G3 samples, it was significantly higher than in G1 samples, suggesting that NRP1 could be a useful prognostic marker [40]. These findings are supported by research conducted by Okon et al., where strong NRP-1 expression was also observed in endometrial cancer, along with an association between NRP1 expression and levels of protumorigenic cytokines [38]. In our study, we found a higher level of NRP1 in the serum of EC patients compared with NCEL patients. These results are significant because most available studies on NRP1 in endometrial cancer focus on its expression in tissues rather than serum. Our discovery of elevated NRP1 levels in the serum of EC patients aligns with tissue study results, suggesting that neuropilin-1 may have significance not only at the tissue expression level but also in the serum. Elevated NRP1 levels in serum may reflect its increased expression in tumor tissue and serve as a useful diagnostic marker for detecting cancer and monitoring its progression.

Our study also has limitations. The study presented here is one of the first to present the diagnostic aspect of the proteins studied in endometrial cancer. Future studies are needed to confirm our observations and better understand the role of the proteins we investigated in the diagnosis of endometrial cancer. Existing literature data confirm the utility of the proteins we assessed in diagnosing and monitoring the progression of endometrial cancer, reinforcing the need for continued research in this area. Further research is needed to validate these findings and explore the underlying mechanisms by which these biomarkers contribute to the pathogenesis of endometrial cancer. Moreover, investigating the potential for combining these biomarkers with existing diagnostic modalities, such as imaging or histopathological assessments, could enhance overall diagnostic accuracy. Understanding the biological roles of these biomarkers in the tumor microenvironment could reveal novel insights into their functions and interactions that influence cancer progression. Additionally, the variability in the levels of these biomarkers among different stages and subtypes of endometrial cancer needs to be explored. This would provide information on whether they have stage-specific or type-specific diagnostic values. The role of these biomarkers in predicting treatment responses and patient outcomes also remains an open question. Potential future research could focus on studies assessing how changes in biomarker level correlate with treatment efficacy and disease progression. Addressing these gaps will be crucial in improving the use of these biomarkers for early detection, prognosis, and personalized treatment strategies in endometrial cancer.

## 5. Conclusions

The initial study presented here demonstrates the clinical utility of IL-4, IL-7, IL-9, NT, and NRP1 in endometrial cancer. IL-4, IL-7, IL-9, NT, and NRP1 may be useful diagnostic markers for endometrial cancer. Perhaps, in the future, these proteins could co-develop a diagnostic protocol to support ultrasound and histological confirmation of endometrial disease. This study showed that IL-10 and TSP-2 should not be used as diagnostic markers in endometrial cancer. In the future, similar studies should be conducted, evaluating combinations of several proteins studied here as diagnostic markers versus single markers. It would also be appropriate to focus on evaluating these proteins as prognostic or predictive markers.

## Figures and Tables

**Table 1:** Characteristics of patients including endometrial cancer characteristics.

Characteristics	Number of Patients (%)
Endometrial cancer	
Yes	93 (58)
No	66 (42)
Endometrial Cancer	
Endometrioid endometrial carcinoma	82 (88)
Nonendometrioid endometrial carcinoma	11 (12)
Clinical staging	
FIGO I and II	88 (95)
FIGO III and IV	5 (5)
Histopathological grading	
Grade 1	37 (40)
Grade 2	34 (37)
Grade 3	22 (24)

**Table 2:** Characteristics of patients including clinical data.

Clinic Demographic Characteristics	Total Cohort (*n* = 159)	Endometrial Cancer (*n* = 93)	NCEL (*n* = 66)	*p*-Value	Statistical Power
**Median (IQR)**					
Age (years old)	55 (43–67)	51 (43–63)	59.5 (43–71)	0.1106	0.995
BMI (kg/m^2^)	27.9 (24.2–31.7)	28.6 (25.4–31.7)	27 (23–31)	0.0397	0.1671
**Number (%)**					
**Age (years old)**					
<65	114 (72)	74 (80)	40 (61)	0.0967	0.999
≥65	45 (28)	19 (20)	26 (39)	0.2889	0.991
**BMI**					
<25	43 (27)	22 (24)	21 (32)	0.5922	0.0947
≥25	116 (73)	71 (76)	45 (68)	0.1176	0.999
**Menopausal status**					
Premenopausal	52 (33)	34 (37)	18 (27)	0.0387	0.999
Postmenopausal	107 (67)	59 (63)	48 (73)	0.3035	0.999
**Hypertension**					
Yes	82 (52)	49 (53)	33 (50)	0.3600	0.999
No	77 (48)	44 (47)	33 (50)	0.5807	0.999
**Diabetes mellitus**					
Yes	73 (46)	42 (45)	31 (47)	0.5577	0.999
No	86 (54)	51 (55)	35 (53)	0.3123	0.999

**Table 3:** Comparison of concentrations of studied proteins between patients with endometrial cancer and noncancerous endometrial lesions.

Characteristics		EC	NCEL	*p*-Value	Statistical Power
IL-4 [pg/mL]	Median	982.36	806.42	0.000023	0.999
	Q1–Q3	855.79–1171.3	679.57–991.25		
IL-7 [ng/L]	Median	190.94	106.79	0.000000	0.999
	Q1–Q3	164.55–232.07	89.9–131.77		
IL-9 [pg/mL]	Median	257.15	198.32	0.000000	0.999
	Q1–Q3	220.92–307.78	163.79–223.46		
IL-10 [pg/mL]	Median	1594.97	3245.68	0.000000	0.999
	Q1–Q3	1257.75–1989.90	2998.79–3889.77		
NT [pmol/L]	Median	367.46	242.33	0.000000	0.999
	Q1–Q3	321.16–461.62	177.79–322.46		
TSP-2 [µg/mL]	Median	88.48	87.6	0.6787	0.0845
	Q1–Q3	71.27–113.41	77.57–109.57		
NRP1 [ng/mL]	Median	40.86	28.74	0.000004	0.999
	Q1–Q3	33.59–48.59	15.68–38.46		

**Table 4:** The correlations between studied proteins, presented as Spearman’s ranges; rs—correlation coefficient.

Variable		IL-4	IL-7	IL-9	IL-10	NT	NRP1
IL-4	rs	1	0.395	0.396	−0.149	0.394	0.304
	*p*-Value	-	0.0000001	0.0000001	0.061206	0.0000001	0.0001
	Statistical power	-	0.979	0.979	0.299	0.978	0.850
IL-7	rs	0.395	1	0.628	−0.420	0.529	0.259
	*p*-Value	0.0000001	-	0.0000001	0.0000001	0.0000001	0.000982
	Statistical power	0.979	-	0.999	0.990	0.999	0.715
IL-9	rs	0.396	0.628	1	−0.229	0.629	0.264
	*p*-Value	0.0000001	0.0000001	-	0.003764	0.0000001	0.000788
	Statistical power	0.979	0.999	-	0.604	0.999	0.732
IL-10	rs	−0.149	−0.420	−0.229	1	−0.348	−0.232
	*p*-Value	0.061206	0.0000001	0.003764	-	0.000007	0.003313
	Statistical power	0.299	0.990	0.604	-	0.934	0.616
NT	rs	0.394	0.529	0.629	−0.348	1	0.251
	*p*-Value	0.0000001	0.0000001	0.0000001	0.000007	-	0.001391
	Statistical power	0.978	0.999	0.999	0.934	-	0.687
NRP1	rs	0.304	0.259	0.264	−0.232	0.251	1
	*p*-Value	0.0001	0.000982	0.000788	0.003313	0.001391	-
	Statistical power	0.850	0.715	0.732	0.616	0.687	-

**Table 5:** Diagnostic values of studied proteins for patients with endometrial cancer.

Marker	AUC (95% CI)	Sensitivity [%]	Specificity [%]	PPV [%]	NPV [%]	*p*-Value	Cut-Off Value
IL-4	0.7 (0.61–0.78)	83.87	50	70.27	68.75	0.000023	802.26 pg/mL
IL-7	0.91 (0.85–0.96)	96.77	75.76	84.91	94.34	<0.000001	133.63 ng/L
IL-9	0.8 (0.73–0.87)	69.89	81.82	84.42	65.85	<0.000001	228.79 pg/mL
IL-10	0.006 (0–0.014)	100	0	58.49	NA	<0.000001	187.4 pg/mL
NT	0.83 (0.76–0.9)	94.62	59.09	76.52	88.64	<0.000001	275.43 pmol/L
NRP1	0.71 (0.63–0.8)	81.72	57.58	73.08	69.09	0.000004	30.37 ng/mL

## Data Availability

The data presented in this study are available from the corresponding author, M.K., upon reasonable request.
